# Anatolian medicinal plants as potential antiviral agents: bridging traditional knowledge and modern science in the fight against COVID-19 and related viral infections

**DOI:** 10.55730/1300-0152.2699

**Published:** 2024-06-26

**Authors:** Engin TİLKAT, Israt JAHAN, Ayşe HOŞER, Alevcan KAPLAN, Oğuzhan ÖZDEMİR, Ahmet ONAY

**Affiliations:** 1 Department of Biology, Faculty of Science and Literature, Batman University, Batman, Turkiye; 2 Department of Health Care Services, Vocational School of Health Services, Mardin Artuklu University, Mardin, Turkiye; 3 Department of Crop and Animal Production, Sason Vocational School, Batman University, Batman, Turkiye; 4 Department of Veterinary Science, Technical Sciences Vocational School, Batman University, Batman, Turkiye; 5 Department of Biology, Faculty of Science, Dicle University, Diyarbakır, Turkiye

**Keywords:** Antiviral plants, COVID-19, MERS-CoV, SARS-CoVs, traditional Anatolian medicine (TAM)

## Abstract

The severe acute respiratory syndrome coronavirus 2 (SARS-CoV-2) was the cause of the coronavirus 2019 (COVID-19), commonly known as the coronavirus pandemic. Since December 2020, COVID-19 vaccines have been extensively administered in numerous countries. In addition to new antiviral medications, the treatment regimen encompasses symptom management. Despite sustained research efforts, the outbreak remains uncontrolled, with affected patients still lacking proper treatment. This review is a valuable asset for researchers and practitioners aiming to delve into the yet unexplored potential of Anatolian flora in the fight against COVID-19 and other viral infections. Numerous medicinal plants in Anatolia, such as thyme, sage, cannabis, oregano, licorice root, and *Origanum* sp., contain bioactive compounds with proven antiviral properties that have been used in the region for centuries. The rich legacy of traditional Anatolian medicine (TAM), has significantly influenced modern medicine; thus, the profusion of medicinal plants native to Anatolia holds promise for antiviral drug development, making this review essential for researchers and practitioners.

## 1. Introduction

The devastating impact of the severe acute respiratory syndrome coronavirus 2 (SARS-CoV-2) on global health, economy, and social life characterizes the coronavirus 2019 (COVID-19) pandemic (Sawicka et al., 2022). Originating in Wuhan, China, in December 2019, the SARS-CoV-2 outbreak rapidly evolved from a local incident to a global crisis within 30 days, underscoring an urgent need for effective strategies and treatments (Zhu et al., 2020). In the 21st century, three coronaviruses, namely the severe acute respiratory syndrome (SARS) coronavirus (SARS-CoV), the Middle East respiratory syndrome coronavirus (MERS-CoV), and the recently emergent SARS-CoV-2, have caused severe pneumonia in humans (Hui et al., 2022). The quest for effective antiviral drugs against these viruses has been emphasized in various studies (Niknam et al., 2022). SARS-CoV-2, in particular, has gained widespread attention, leading to extensive research efforts to discover effective therapeutic interventions (Pribanić et al., 2022). Oxygen therapy and corticosteroid dexamethasone have shown efficacy in treating severe cases (Patil et al., 2023).

While several drugs have been approved and utilized, including Remdesivir and monoclonal antibody drugs, challenges such as emerging resistance and safety concerns persist (Razonable et al., 2021). The importance of immunization, highlighted by the World Health Organization (WHO), has been underscored with ongoing research exploring additional therapeutic strategies for COVID-19 (Almuqbil et al., 2023). However, the dynamic nature of the virus, marked by its mutations, necessitates ongoing efforts to develop effective prevention and control measures (Zhao et al., 2021). Recognizing the limitations and challenges of current approaches, some researchers have turned to plant-based medicinal alternatives, leveraging the bioactive components of herbs and plants with known antiviral properties (Al-Kuraishy et al., 2022). In this context, traditional medicinal systems, particularly herbal-based ones, have gained attention for their potential in combating viral infections (Yasmin et al., 2020; Elfiky, 2021).

Traditional Anatolian medicine (TAM), with its rich heritage and centuries-long influence on modern medical practices, presents a promising avenue for exploring antiviral solutions (Sezik et al., 1997). Anatolia’s diverse topography and climate have fostered a unique botanical tradition, with numerous plant species exhibiting medicinal properties (Demir and Demir, 2022). Of particular interest are medicinal plants, such as thyme, sage, oregano, licorice root, and others, known for their bioactive compounds with antiviral potential (Abiri et al., 2021). Therefore, this review aims to delve into the antiviral properties of Anatolian medicinal plants and underscore their significance in the development of broad-spectrum antiviral drugs, including those targeting the root pathogens of COVID-19. By understanding the antiviral characteristics of these plants and fostering collaboration among traditional medicine practitioners, ethnobotanists, pharmacologists, and virologists, we can better prepare for future virus outbreaks and safeguard global health.

## 2. Methodology

This review employed a comprehensive methodology to gather relevant data on SARS-CoV-2, COVID-19, and past pandemics. Reputable scientific databases, including PubMed, ScienceDirect, Springer, and Elsevier, were systematically searched from January 2020 to July 2023 to incorporate the latest findings. Additionally, information from books and previously published research articles was scrutinized. To ensure a thorough examination, databases such as DergiPark (TÜBİTAK ULAKBİM) were utilized to explore traditional Anatolian medicine. Keywords such as ‘Covid-19’, ‘SARS-CoV-2’, ‘antiviral plants’, ‘traditional medicine’, ‘Turkish medicine’, and ‘Anatolian medicinal plants’ were strategically employed during the search strategy.

The initial search yielded 2698 articles, which underwent screening based on titles, abstracts, and complete texts to identify studies relevant to the review’s focus. A broad range of search terms, including ‘epidemic’, ‘flu’, ‘pandemic’, and ‘bioactive compounds’, was applied to explore potential connections between SARS-CoV-2 and TAM. This approach aimed to elucidate the relationships between traditional Anatolian medicinal plants and their bioactive compounds within the context of epidemics and pandemics.

Data extraction and synthesis were meticulously conducted to present coherent insights and draw conclusions from the selected studies. The research underwent a rigorous reliability and validity assessment to ensure high-quality information. This review aims to offer important insights into the potential of herbal folk medicines from Anatolia as alternative sources for developing antiviral treatments, focusing on COVID-19 and related viral infections.

## 3. Origin of COVID-19

Coronaviruses, recognized for their high pathogenicity among birds and mammals, belong to the *Coronaviridae* family in the *Nidovirales* order (Woo et al., 2010). Genomic analysis suggests that, like other SARS viruses, SARS-CoV-2 likely originated in bats, which served as the primary reservoir and natural host. However, the complete zoonotic transmission process and the intermediary source of the virus’s transfer to humans remain uncertain (Liu et al., 2020). There is speculation suggesting that snakes and pangolins may have functioned as intermediate hosts, facilitating the transmission of this pathogen (Zhang et al., 2020a). In 2002, Guangdong Province in China witnessed the emergence of a SARS-CoV outbreak that led to atypical pneumonia (Feng et al., 2009). The virus swiftly spread across several countries, impacting over 8000 individuals (Peiris et al., 2004). In 2012, a separate coronavirus, MERS-CoV, surfaced in Saudi Arabia, resulting in severe respiratory illnesses and numerous fatalities (Madabhavi et al., 2020).

The origins of COVID-19 can be traced back to patients experiencing severe respiratory problems admitted to hospitals in Wuhan, China, in late 2019 (Sun et al., 2020). The initial cases were tied to the trading of live animals at the Huanan Seafood Market in Wuhan, with human-to-human transmission already underway (Hu et al., 2021). On February 11, 2020, the WHO officially designated the illness COVID-19 caused by SARS-CoV-2. Genetically, it is related to SARS-CoV and pangolin and bat coronaviruses (Perlman, 2020; Zhang et al., 2020b). The first reported fatality linked to COVID-19 occurred in China on January 9, 2020, signaling the beginning of a swift worldwide transmission of the virus. As of January 30, 2020, there were 7818 confirmed cases across 19 countries, prompting the WHO to announce a “Public Health Emergency of International Concern.”^1^

As of March 11, 2020, after over 118,000 cases had been reported across 114 countries, the situation was officially classified as a “pandemic” by the WHO^1^. As of August 12, 2023, the global count had surged to 769,774,646 confirmed COVID-19 cases, with 6,955,141 fatalities. The profound impact of this pandemic, reflected in its devastating fatality toll, firmly establishes it as one of the deadliest events in recorded human history^2^.

### 3.1. COVID-19 and traditional herbal therapy

Since ancient times, plants have been crucial in global drug production and managing infectious diseases. In contrast to synthetically developed antiviral drugs, which may carry potential side effects and health risks, herbal-based medicines offer a promising avenue to mitigate these concerns by providing effective antiviral potential. Herbal extracts, encompassing essential oils and secondary metabolites, may contain active compounds with anti-COVID-19 properties (Jahan and Onay, 2020). With the emergence of the COVID-19 pandemic, a growing focus on traditional Chinese herbal medicinal practices materialized. These approaches included oral administration of preservative herbal formulas, combining herbal medicine with commercial ones for prevention and treatment, and indoor herbal medicine fumigation. The renewed interest in traditional Chinese medicine was sparked by its proven effectiveness during the SARS outbreak in 2003 (Jahan and Onay, 2020).

Natural compounds found in various herbs have been recognized for their antiviral potential, frequently pinpointing distinct stages of viral replication and infection since 2003. Generations of traditional knowledge have highlighted herbs known for their immune-boosting, antiinflammatory, and antiviral properties (Jahan and Onay, 2020). Plant-derived chemicals like flavonoids, polyphenols, and essential oils have shown promise in combating various viruses (Abiri et al., 2021). Traditional herbal therapy offers a promising way to boost the immune system and potentially stop the spread of viruses, such as SARS-CoV-2 and others, by utilizing these innate defenses. As we explore the intersection of COVID-19 and traditional herbal therapy, diverse possibilities emerge that could enrich our defenses against viral pandemics. This scholarly endeavor involves a meticulous compilation and analysis of a diverse spectrum of data sources of paramount significance in uncovering potential solutions in the fight against viral outbreaks (Jahan and Onay, 2020).

### 3.2. COVID-19 treatment potential of TAM

Exploring the potential use of herbs from TAM in treating COVID-19 is a crucial endeavor. Many plant species in Türkiye, especially in the Anatolian region, have therapeutic qualities and have been used for millennia in traditional medicine. These plants harbor many secondary compounds, boasting antiviral, anticancer, and diverse therapeutic properties, making them promising resources for potential treatment options (Sezik et al., 1997). Plants native to Anatolia could be foundational in addressing various ailments, including COVID-19. The information presented in Table highlights a range of medicinal plants from Anatolia with antiviral properties and active compounds, providing a valuable resource for potential COVID-19 treatments.

#### 3.2.1. *Allium* L

The *Allium* genus, a prominent member of the *Liliaceae* family, encompasses around 500 species worldwide (Fenwick and Hanley, 1985). There are 190 identified species of *Allium* L. in Turkish flora, 75 of which are indigenous (Eker and Koyuncu, 2011). This genus has attracted considerable attention due to its diverse pharmacological potential, encompassing attributes such as antitumoral (Asemani et al., 2019), antimicrobial (Barbu et al., 2023), antioxidant (Rocchetti et al., 2022), antiviral (Lengbiye et al., 2020), and antidiabetic (Sabiu et al., 2019) properties. Highlighting the significant potential of *Allium* species in addressing viral infections, including the SARS-CoV-2 pandemic, is of considerable importance. Preliminary research has indicated that specific bioactive compounds found in *Allium* plants may demonstrate inhibitory effects against SARS-CoV-2, rendering them promising candidates for developing antiviral therapeutics (Rouf et al., 2020). However, comprehensive studies are needed to ascertain specific mechanisms of action, dosage requirements, and potential synergistic effects with existing antiviral treatments. Exploring the potential role of the *Allium* genus in addressing antiviral activities against viruses, including SARS-CoV-2, could pave the way for novel and effective treatment strategies for emerging virus-based global health crises.

Türkiye has a variety of wild and endemic species of garlic (*Allium sativum* L.), along with many other endemic species. For example, Türkiye’s Tunceli Province is home to *Allium tuncelianum* (Kollmann) Özhatay, B. Mathew & Siraneci, often known as Tunceli garlic. Another example is *A. macrochaetum* Boiss. and Hausskn., called rock garlic, found in the heart of the Eastern Anatolia region (Şenkul and Kaya, 2017). Additionally, *Allium flavum* (yellow garlic) subspecies flourish in Turkish regions, such as *A. flavum* var. minus Boiss. and *A. flavum* subsp. *flavum* in the Uludağ region of Bursa, and *A. flavum* var. *pilosum* Kollmann and Koyuncu in the Adana Province of Türkiye. *Allium kurtzianum* Asch. & Sint. ex Kollmann, an endangered wild garlic, is native to the Kaz mountain range in Western Türkiye (Çanakkale) (Torlak et al., 2010).

However, there is no contemporary scientific backing for the therapeutic benefits of many of these garlic variants. Nonetheless, the health advantages of garlic are attributed to its sulfur-containing constituents, including S-allyl cysteine and allicin (Sobenin et al., 2019). Among these organosulfur compounds, allicin (diallyl thiosulfate), ajoenes, and thiosulfinates play fundamental roles in treating numerous illnesses (Suleria et al., 2015). Notably, the pharmacological effects of allicin, a key compound in garlic, are only present when garlic is crushed. The allicin compound forms due to the interaction of the alliinase enzyme and alliin within the garlic tissue, a process triggered by crushing, cutting, or chewing. Another influential compound is ajoene, which results from enzymatic breakdown (Blania and Spangenberg, 1991). The impact of bioactive compounds like alliin (S-allyl-L-cysteine sulfoxide) and diallyl sulfide on adenovirus-3 and coronavirus (CoV) has been studied, revealing significant reductions in disease efficiency, particularly in SARS-CoV (Rouf et al., 2020). Given the life-threatening immunosuppressed state of COVID-19 patients, the immune-boosting properties of garlic are potentially significant. Akram et al. (2021) reported that the consumption of garlic possibly mitigates certain clinical symptoms, including the loss of taste associated with COVID-19.

The garden onion (*Allium cepa* L.) harbors a unique combination of phytochemicals. This includes flavonoids like quercetin and kaempferol, phenolic acids, and biologically functional sulfur-containing compounds, such as sulfonic acids and thiosulfinates (Nishimura et al., 2019). Furthermore, it contains vital nutrients like potassium, selenium, vitamins B1, B2, and C, proteins, and saccharides (Nishimura et al., 2019). Numerous studies have confirmed the inhibitory potential of various therapeutic substances found in onions, such as quercetin, kaempferol, zalcitabine, allicin, and ribavirin, against adenovirus (Sharma, 2019). Epidemiological and in vitro/in vivo animal model studies have suggested that the Welsh onion (*Allium fistulosum* L.) has defensive capabilities against various illnesses, spanning respiratory (e.g., bronchitis, fever, cold, dyspnea), gastrointestinal (e.g., dysentery), and cardiovascular (e.g., angina pectoris) disorders (Lee et al., 2012). Additional studies, including those involving mice, have indicated that onion consumption is linked to increased mineral density and bone mass, potentially mitigating the risk of osteoarthritis. Human studies have suggested that daily onion consumption leads to a 5% greater bone density than if consumed once a month or less (Matheson et al., 2009).

#### 3.2.2. *Salvia* L

*Salvia*, also known as sage, is the largest genus in the *Lamiaceae* family, boasting an impressive 1000 species (Walker et al., 2004). Türkiye is home to a diverse collection of the *Salvia* genus, encompassing over 90 species and 94 taxa (Kurkcuoglu et al., 2019). *Salvia* species show promise in preventing conditions such as hypoxia, viral infections, oxidative damage, and Alzheimer’s, among other health benefits (Lopresti, 2017). These species are especially high in rosmarinic acid, which by itself exhibits a variety of beneficial benefits, including antioxidative, antiinflammatory, antimutagenic, antimicrobial, antibacterial, and antiviral characteristics (Yılmaz et al., 2017). Conducting molecular docking experiments, the effectiveness of rosmarinic acid against SARS-CoV-2 was evaluated by targeting both the spike (S) and major protease (M^pro^) receptors (Elebeedy et al., 2021). Additionally, *Salvia fruticosa*, found in the same botanical family, has been proven to contain potent antiviral properties. The use of CAPeo, utilizing essential oils derived from this species, led to a decrease in the production of offspring viruses for both human rhinovirus 14 (HRV14) and influenza A/H1N1 viruses (Tseliou et al., 2019). An unexpected discovery brought attention to the powerful antiviral properties of crude *S. officinalis* extract against the avian influenza H5N1 virus and the vesicular stomatitis virus, as highlighted by Abou-Baker et al. (2021). Furthermore, Ezema et al. (2022) explored the medicinal potential of the *Salvia* species in treating cancer and viral infections.

#### 3.2.3. *Sideritis* L

The *Sideritis* genus, a prominent *Lamiaceae* (*Labiatae*) family member, encompasses over 319 distinct species, rendering it one of the most expansive and diverse plant genera. These flowering plants are known as shepherd’s tea, mountain tea, or ironwort (Barber et al., 2000). Anatolia is one of two genetic centers of the *Sideritis* species; this remarkable genus boasts an impressive endemism rate of nearly 80% and a spectrum of 46 *Sideritis* species (52 taxa). Among these, *Sideritis brevibracteata* P.H. Davis, colloquially known as “Mountain tea” or “Alanya sage,” thrives predominantly in the Alanya/Antalya region (Sagir et al., 2017).

For centuries, *Sideritis* plants have played a crucial role in Turkish traditional and folk medicine as remedies for colds, flu, respiratory issues, allergies, and digestive ailments (González-Burgos et al., 2011; Ertas and Yener, 2020). These plants exhibit remarkable antiulcerogenic, antiinflammatory, antimicrobial, and antiviral properties (Obón et al., 2021). Furthermore, extracts derived from *Sideritis* spp. have been identified as containing linearol and its derivatives, showcasing notable antiviral effects against HIV (Abeshi et al., 2017). Previous analysis has revealed active components such as terpenes, phenolics, coumarins, and lignans in the plant extracts and essential oils of *Sideritis* spp. (Sagir et al., 2017). During the pandemic, many people in Central and Eastern Anatolia adhered to traditional practices, relying on *Sideritis* spp. to address not only fevers, sore throats, and coughs but also various other symptoms associated with COVID-19 (Akbulut, 2021). Moreover, recent research has highlighted the potential of ent-kaurane diterpenoids, such as linearol, sidol, and isosidol from the acetone extract of the *S.lycia* species, which have demonstrated strong antiviral activities (Kılıç et al., 2020). As a result, the *Sideritis* species are promising candidates for future therapeutic applications and warrant further comprehensive exploration.

#### 3.2.4. *Thymus* L

Within the *Lamiacea*e family, the *Thymus* genus is botanically remarkable, boasting an astounding 53% endemism rate and encompassing a rich tapestry of 39 species and 59 taxa across Türkiye (Karaman et al., 2001). Thyme oil is ubiquitous in medicinal formulations and is also used in antibacterial remedies. The essential oil derived from *T. vulgaris* has been traditionally employed in rural practice as a remedy for inflammation-related conditions, such as rheumatism, muscle edema, insect bites, and other bodily discomforts. It is also utilized for treating pruritus associated with dermatitis, bruises, sprains, parasitic infections, coughing, symptoms of bronchitis, upper respiratory tract infections, digestive disorders, and menstrual problems (Hosseinzadeh et al., 2015; Kowalczyk et al., 2020). Furthermore, the many active ingredients in thyme oil have also been shown in contemporary medicine to provide a range of advantages, including antimicrobial, antiviral (Newcastle disease virus, influenza viruses, human rhinoviruses, and herpes simplex virus type 1 and 2), antifungal, antioxidant, and antiinflammatory effects (Dursun et al., 2003; Salehi et al., 2018a; Kowalczyk et al., 2020; Tohidi et al., 2020; Catella et al., 2021). In addition, contemporary research has emphasized carvacrol as a potential candidate for use with other preventive or therapeutic measures to address SARS-CoV-2. This substance exhibits potent antiinflammatory, antiviral, and immunomodulatory activities, which could mitigate the severity and duration of the illness (Javed et al., 2021). A detailed molecular review revealed the capacity of these effects to suppress inflammatory cytokines, boost antiinflammatory factors, and positively impact the renin-angiotensin system and intestinal microbiota. These promising results point to potential in preventing COVID-19 complications and alleviating its effects (Nadi et al., 2023). The remarkable prospect of the *Thymus* genus in treating COVID-19 was further supported by an in silico analysis demonstrating the prospective inhibitory effects of various essential oil components extracted from *T. schimperi* on the SARS-CoV-2 main protease (Mengist et al., 2023). Although *T. schimperi* is currently exclusive to Ethiopia as an endemic species, the tantalizing prospect exists that other members of the same genus, found within Anatolia’s unique endemic flora, may exhibit antiviral abilities comparable to or even superior to those of this particular species.

#### 3.2.5. *Origanum* L

The *Origanum* species are taxonomically classed as herbaceous perennials, subshrubs, and fragrant plants, all belonging to the *Lamiaceae* family (Chávez-González et al., 2016). The most often-used species in this genus is *Origanum vulgare* L., often known as “oregano” in many countries (Skoula and Harborne, 2002). Within the Turkish flora, 31 taxa of this genus, encompassing 27 species, have been documented and utilized as food additives and traditional remedies for long periods dating back to the Hittite era (1600–1200 BC) (Celep and Dirmenci, 2017).

Recent research has suggested that the specific essential oils of oregano might impact the SARS-CoV-2 delta variant by inhibiting the virus’s cellular entry in vitro (Sargin, 2021; Torres Neto et al., 2022). Likewise, emerging research on the medicinal qualities of carvacrol, a monoterpene phenol prevalent in oregano oils, has demonstrated its potential as a promising treatment option for viral infections, including COVID-19, owing to its diverse biological and pharmacological properties (Javed et al., 2021). However, further investigation is needed to validate these phytoconstituents’ anti-SARS-CoV-2 effectiveness in vitro and in vivo, utilizing relevant animal and cell culture models (Husain et al., 2022).

Research on the interactions of essential oils with viral proteins has continuously demonstrated the antiviral efficacy of essential oils extracted from various *Origanum* species, including *Origanum vulgare*. Additionally, a recent study has shown the antiviral efficacy of essential oil blends derived from a range of aromatic plants, such as *Salvia fruticosa* Mill, *Origanum dictamnus* L., and *Coridothymus capitatus* L. Influenza A/H1N1 virus strains and HRV14 were among the respiratory viruses against which the study found that the essential oil mix had shown remarkable antiviral efficacy. This implies that essential oils like those found in *Origanum* spp. may have antiviral activities against SARS-CoV-2 comparable to those of these oils (Tseliou et al., 2019). Additional research is necessary to fully understand the specific mechanisms and potential applications of *Origanum* essential oils in treating viral diseases like SARS-CoV-2.

#### 3.2.6. *Glycyrrhiza* L

The *Fabaceae* family includes *Glycyrrhiza* L., also known as licorice. Found throughout the eastern Mediterranean region of Türkiye, it is well-known for its therapeutic uses in TAM (Altay et al., 2016). Licorice is considered an important herb because of its widespread use in treating illnesses such as colds, immunodeficiency, cancer, and atherosclerosis. It has increasingly been used in traditional herbal medicine around the world. Scientific investigation has highlighted its significant role in managing various health conditions (Altay et al., 2016).

**Figure 1:**
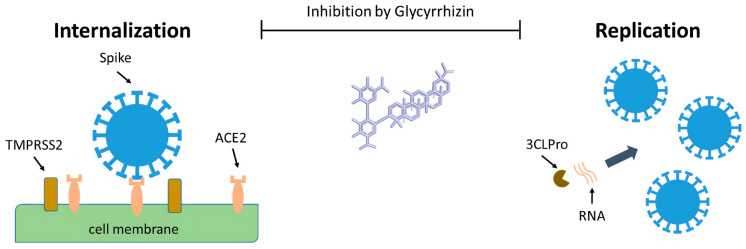
Potential impact of glycyrrhizin on the uptake and multiplication of the SARS-CoV-2 virus in the host cell (adapted from Diomede et al., 2021). (Source: This open-access article is distributed under the Creative Commons Attribution 4.0 License.)

**Figure 2:**
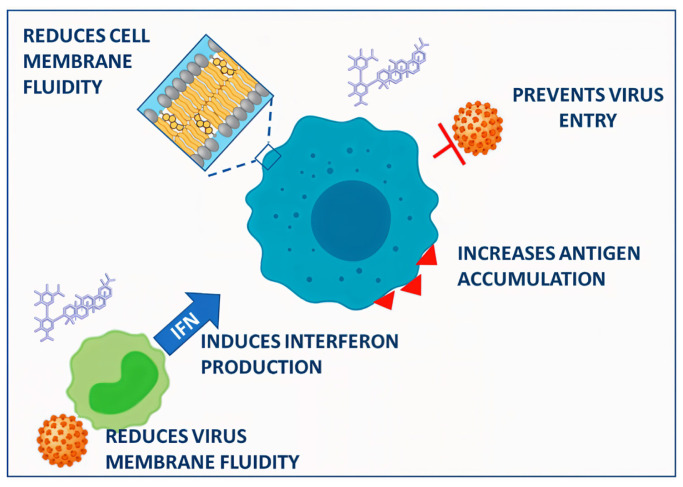
Proposed mechanisms for how glycyrrhizin works as an antiviral (adapted from Diomede et al., 2021). (Source: This open-access article is distributed under the Creative Commons Attribution 4.0 License.)

Recent research has demonstrated its potent antiviral efficacy, especially against SARS-CoV-2, the virus that sparked the COVID-19 pandemic (Sinha et al., 2020). The main active ingredient of licorice root, glycyrrhizin (GR), has been shown to dramatically inhibit SARS-CoV-2 reproduction by specifically targeting the main viral protease (M^pro^). This enzyme is essential for viral replication. Given that glycyrrhizin-containing products may be beneficial for treating SARS-CoV-2 infection in COVID-19 patients, these results suggest that further investigation into the potential clinical use of such products is required. Black licorice and licorice root tea are two examples (Diomede et al., 2021; Işık et al., 2022). The antiviral potential of *Glycyrrhiza glabra* (licorice) extract has received much attention recently, especially in the context of COVID-19. This potential is principally attributable to GR and glycyrrhetinic acid (GA). In silico studies have suggested interactions with key viral components, and clinical trials have since been initiated. The results have highlighted the diverse pharmacological effects of GR and GA, encouraging future investigation of their potential contribution to the prevention of COVID-19 and SARS-CoV-2 (Diomede et al., 2021). The spike protein attaching to the ACE2 receptor is how the SARS-CoV-2 virus penetrates host cells (Wu et al., 2021; Lan et al., 2020). A direct relationship between GR and GA and important viral elements involved in internalization and replication, such as ACE2, spike protein, and its receptor-binding domain (RBD), and 3CL^pro^, has been shown in in silico research (Figure 1) (Diomede et al., 2021). This phenomenon is explained by GR’s ability to interact with cellular membranes, which causes a reduction in membrane flexibility and a positive surface charge (Figure 2). A recent study has uncovered potential antiviral medicines associated with GR that may aid in the fight against the COVID-19 pandemic (van de Sand et al., 2021).

The study concentrated on GR, and the researchers assessed how effective this ingredient could be against the SARS-CoV-2 virus using computational and in vitro techniques. According to the study, licorice chemicals show a significant affinity for the virus’s M^pro^, which may prevent the virus from replicating. Further molecular docking and simulation experiments supported these findings. In addition, in vitro tests demonstrated that GR, which is more potent than licorice, exhibits strong anti-SARS-CoV-2 effects. The study presented licorice and GR as interesting options for COVID-19 therapy and urged additional exploration through in vivo experimentation and clinical trials (Tolah et al., 2021). Finally, research into the antiviral properties of *Glycyrrhiza glabra* in relation to SARS-CoV-2 has increased due to the properties found in licorice. GR and GA’s capacity to prevent viral replication, especially in the case of SARS-CoV-2, points to a crucial area for prospective future clinical studies and therapeutic approaches (van de Sand et al., 2021). These findings highlight the significance of additional research into *Glycyrrhiza glabra*’s function in the fight against COVID-19 and its potential to create additional antiviral options.

#### 3.2.7. *Mentha* L

The *Mentha* genus, a member of the *Lamiaceae* or mint family, can be divided into 42 species, 15 hybrids, and hundreds of subspecies, variations, and cultivars found worldwide (Salehi et al., 2018b). This group has a longstanding history of culinary and medicinal use, offering a range of benefits, from being a digestive aid to helping with cognitive enhancement (Anwar et al., 2019). In Türkiye, spearmint (*Mentha spicata*) is used as a flavoring agent in salads and herbal teas and favored in traditional folk medicine to address stomach ailments, flu, and colds (Sevindik et al., 2017). Intriguingly, the biochemical composition of *Mentha* plants has revealed unique bioactive compounds that exhibit potential antiviral effects, notably against avian influenza (Barbour et al., 2010). Significantly, mint’s antiviral capabilities also extend to COVID-19 (Wang et al., 2020).

Amid the ongoing global COVID-19 challenge, the spotlight has turned to *Mentha* L., also known as peppermint or mint, as a potential antiviral resource. Serlahwaty and Giovani’s (2021) in silico study focused on *Mentha piperita* L. leaves, delving into interactions between mint’s active compounds like rutin, hesperidin, and isorhoifolin and the SARS-CoV-2 receptor protein 5R7Y. By revealing the prospect of mint leaf compounds exerting antiviral effects against SARS-CoV-2 receptors, this research exemplifies the ongoing exploration of natural sources for antiviral candidates, contributing to the broader quest for effective COVID-19 treatments.

Recent studies have highlighted the antiviral qualities of essential oils originating from the *Lamiaceae* family, including several *Mentha* species. Ćavar Zeljković et al. (2022) conducted a groundbreaking in vitro study elucidating the antiviral activity of selected *Lamiaceae* essential oils and their monoterpenes against SARS-CoV-2. Their investigation encompassed 19 essential oils obtained from hydrodistillation and identified several active monoterpenes. These compounds, including carvacrol, carvone, and pulegone, inhibited SARS-CoV-2 replication in infected cells. The research highlights the potential of mint-related constituents as agents against SARS-CoV-2 and underscores their significance in pursuing effective therapeutic interventions.

#### 3.2.8. *Primula* L

*Primula* L., commonly known as primrose, belongs to the *Primulaceae* family and comprises low-growing perennials with basal leaves and distinctive five-petaled flowers of various colors. In Türkiye, *Primula* species thrive in diverse habitats, mainly mountainous and moist areas. Notable species like *Primula vulgaris*, *Primula veris*, and *Primula elatior* are prevalent in Northwestern and Northeastern Türkiye and benefit from the nation’s diverse microclimates.

Recent studies have indicated that *Primula* L. is effective against SARS-CoV-2. A study by Tran et al. (2022) showed primrose (*Primula veris* L.) extract’s notable effects; the association between the SARS-CoV-2 spike protein and the ACE2 receptor was seen to be inhibited by substances such as IMU (immunomodulatory) and Bronchipret thyme-primrose (BRO-TE), while Bronchipret thyme-primrose (BRO TP) showed an increase in the release of antiviral substances, suggesting immune support potential. Another pilot investigation by De Pellegrin et al. (2021) investigated the antiviral activity of herbal extracts against SARS-CoV-2. The in vitro SARS-CoV-2 RNA load was significantly reduced in BRO TP extract and partially suppressed by IMU and TOP (thyme-oregano-primrose). These results stress the need for more clinical investigation and highlight BRO TP extract’s remarkable antiviral effectiveness. Furthermore, primulic acid, a saponin found in select *Primulacea*e species, including *Primula veris* L., has shown potential inhibitory effects on SARS-CoV-2. Molecular docking studies conducted by Bilginer et al. (2022) revealed that primulic acid exhibited promising binding affinity to the COVID-19 M^pro^. This aligns with prior indications of antiviral activity associated with primulic acid (Helal and Melzig, 2011). *Primula veris* L., traditionally used for respiratory conditions, is relevant in terms of COVID-19’s respiratory impact. While in vitro investigations are necessary for validation, previous findings underscore the potential of primulic acid and *Primula veris* in contributing to anti-SARS-CoV-2 treatments.

#### 3.2.9. *Prunella* L

*Prunella* L., known as a self-heal or heal-all, shows promise as a COVID-19-causing SARS-CoV-2 inhibitor. This genus includes well-known species, including *Prunella vulgaris* L., *P. orientalis* L., *P. laciniata* L., *P. grandiflora* L., and other variants; these have a broad distribution encompassing Europe, the Mediterranean basin, North Africa, Russia, East Asia, America, and Australia. Türkiye is home to several *Prunella* species, including *P. vulgaris* L., *P. orientalis* L., and *P. laciniata* L., which increases the plant’s regional significance (Ahmed and Ezer, 2008).

The inhibitory ability of *Prunella vulgaris* extract on SARS-CoV-2 viral entrance was examined by Ao et al. (2021). The researchers showed that an aqueous extract from *Prunella vulgaris* had strong inhibitory effects against SARS-CoV-2 spike protein-mediated infections using a sophisticated system incorporating a pseudotyped HIV-1-based vector. Notably, the virus’s mutant and wild-type forms were inhibited by this action. The SARS-CoV-2 spike protein’s ability to bind to its receptor ACE2 was observed as being disrupted by the extract—called NhPV—and it prevented the first stages of viral entry. This study emphasizes the capacity of *Prunella vulgaris* to target several viral types and its potential as an antiviral drug against SARS-CoV-2.

Yang et al. (2023) investigated the processes behind the potential of *Prunella vulgaris* L. as a botanical medicine against COVID-19-associated acute kidney injury (COVID-19 AKI). The authors found important target genes and active chemicals inside *Prunella vulgaris* that may help to explain its protective benefits in network pharmacology and bioinformatics investigations. The research showed that *Prunella vulgaris* chemicals, particularly quercetin, luteolin, and kaempferol, successfully bind to proteins involved in critical signaling pathways, such as NF-B. These findings shed light on the possible use of *Prunella vulgaris* as a treatment for COVID-19 AKI by indicating that it may exert its protective effects by altering inflammatory pathways. Overall, a growing body of research focuses on the potential of *Prunella* L. extracts, notably *Prunella vulgaris*, as effective tools against SARS-CoV-2 infection and its consequences. However, more research is required to clarify the underlying mechanisms and confirm the effectiveness of *Prunella* L. as a potential botanical treatment for COVID-19.

#### 3.2.10. *Malva* L

*Malva*, a genus in the *Malvaceae* family, has gained interest because it may have inhibitory effects on COVID-19’s causative virus, SARS-CoV-2. There are roughly nine species of this genus found in Türkiye alone, and they are widely dispersed over tropical and temperate climates (Davis, 1966; Cullen, 1967). *Malva sylvestris*, often known as common mallow, has been substantially researched to determine its bioactive components and therapeutic potential (Silveira et al., 2020). It is renowned for its exceptional antioxidant, antiinflammatory, antimicrobial, and hepatoprotective qualities. The historical use of this plant in conventional treatment and pharmaceutical formulations highlights the species’ potential as a valuable source of herbal medicine.

Its multifaceted health benefits, including metabolites, antioxidants, and applications against inflammation and cancer, offer insight into this natural therapeutic agent’s origins (Mousavi et al., 2021). The rich phytochemical content of *Malva sylvestris*, which includes polyphenols, flavonoids, and tannins, has shown promise in suppressing SARS-CoV-2 (Irfan et al., 2021). *Malva sylvestris* extracts in various forms have demonstrated strong antioxidant activity on par with well-known antioxidants. The dichloromethane extracts exhibited high antiradical activity against DPPH and NO radicals. In silico studies and bioassays have highlighted the antioxidant properties of *Malva sylvestris*, indicating its potential as an adjuvant therapy tool against COVID-19 (Silveira et al., 2020). The plant’s high safety profile makes it a valuable herbal medicine for alleviating COVID-19 symptoms and improving patient well-being. However, further exploration is necessary to fully harness its benefits by investigating its active compounds and mechanisms. It is worth noting that the phytochemicals found in *Malva neglecta* Wallr, a species in the *Malva* genus with strong antioxidant capacities (Dalar et al., 2012), may also have the ability to act as an effective drug against COVID-19. These polyphenolic compounds, isolated from plant extracts, have shown antiviral effects by inhibiting crucial proteins necessary for virus infection and replication. Their use offers the advantage of promoting patient well-being with minimal side effects. Molecular descriptors, electrostatic potential, and molecular orbitals were studied to understand their antiviral behavior. Through molecular docking, these phytochemicals were found to inhibit the core protease protein (6LU7) of COVID-19, suggesting their potential as antiviral agents (Irfan et al., 2021.)

#### 3.2.11. *Rosmarinus* L

Three wild plants of the genus *Rosmarinus* (*Lamiaceae*)—*Rosmarinus officinalis*, *Rosmarinus eryocalix*, and *Rosmarinus tomentosus*—grow primarily in the western Mediterranean region. A fragrant evergreen plant native to the Mediterranean area, rosemary (*Rosmarinus officinalis* L.) is a member of the *Lamiaceae* (mint) family (Andrade et al., 2018). *R. officinalis* grows in Türkiye from just above sea level to 1000 m on scrublands, hillsides, and dry, rocky slopes in pine forests, especially in the Mediterranean region (Binzet et al., 2020). Due to its significant medicinal potential, research interest has focused on the plant’s tall, slender leaves and blue, purple, pink, or white blooms. Proponents of traditional medicine recommend taking rosemary orally to treat dysmenorrhea, renal colic, and muscle spasms. Essential bioactive substances, primarily phenolic and terpenes, such as α-pinene, camphor, 1, 8-cineol, and bornyl acetate, are found in the plant (Soncu et al., 2020). In addition to their antiviral, antioxidant, antifungal, antiulcerogenic, antidepressive, antithrombotic, anticancer, antiinflammatory, antibacterial, and antinociceptive capabilities, these chemicals support a wide range of biological activities (AL-Megrin et al., 2020). The therapeutic properties of *R. officinalis* continue to be used to treat a wide range of illnesses, including those affecting the reproductive, gastrointestinal, neurological, hepatic, genitourinary, cardiovascular, and menstrual systems. Additionally, it eases respiratory problems as well as skin discomfort. According to recent research, rosemary’s rosmarinic acid content has antiviral properties that can work against the herpes simplex (HSV-1 and HSV-2) virus (AL-Megrin et al., 2020).

This inherent quality emphasizes how effective it could be as an antiviral medication. In addition to being used medicinally, rosemary has been widely used in the food and cosmetic industries due to its numerous advantages. Additionally, according to recent computational studies, bioactive polyphenols and terpenoids from rosemary may block the SARS-CoV-2 M^pro^, a crucial actor in viral replication (Patel et al., 2023). Furthermore, recent studies have demonstrated that rosemary essential oil (*R. officinalis* L.) and its main component, 1,8-cineole, could be useful against SARS-CoV-2. The essential oil showed notable 5-LOX inhibition (81.1%) and significant ACE2 inhibition (20%) due to its high concentrations of 1,8-cineole (62.7%), α-pinene (12.6%), and camphor (8.3%) (Demirci et al., 2022). Notable ACE2 inhibition (89.2%) and 5-LOX inhibition (37.2%) both result from 1,8-cineole (Demirci et al., 2022). The potential of rosemary essential oil and its main ingredient in becoming part of antiviral methods, particularly against coronaviruses, is highlighted by the suppression of ACE2 and its antiinflammatory effects (Demirci et al., 2023). Although these in vitro results show promise, more in vivo research is required to confirm the therapeutic efficacy of rosemary essential oil and its constituent parts. This implies that rosemary has a wide range of applications, including in the treatment of viral infections, highlighting the importance of this plant in the ongoing search for alternative COVID-19 medicines. To completely understand rosemary’s role in preventing SARS-CoV-2 infections, more research is required, both in vivo and in simulation.

#### 3.2.12. *Pistacia* L

The *Pistacia* genus, a member of the *Anacardiaceae* family, comprises 12 distinct taxa, including both species and subspecies (Nezami and Gallego, 2023). Practitioners of traditional medicine have long utilized various parts of the *Pistacia* species to combat a range of ailments, using them as tonics, aphrodisiacs, diuretics, and antiseptics; they are also known for their effectiveness against gastrointestinal, urinary, and respiratory tract disorders and dental and gum diseases (Tilkat et al., 2018). Scientific research has described their diverse uses and antiviral, antimicrobial, antioxidant, antiinflammatory, anticholinesterase, antitumor, and antidiabetic pharmacological activities—and their benefits against gastrointestinal disorders (Tilkat et al., 2018; Ayaz Tilkat et al., 2021; Rauf et al., 2022; Tilkat et al., 2023). Of note are the linoleic and palmitic acids found in *Pistacia vera* L. oil extract, which are known to contain strong antiviral effects against HSV-1 and parainfluenza viruses (Özçelik et al., 2005).

Recent studies have also looked into the potential of *Pistacia integerrima* and *Pandanus odorifer* plant extracts as efficient inhibitors against the RBD of the spike protein of SARS-CoV-2, highlighting their antioxidant properties and bioactive compounds (Paul et al., 2022). In particular, *Pistacia lentiscus* bark has exhibited significant antimicrobial and antioxidant activities against bacteria and yeast, suggesting its potential use as an antimicrobial and antiviral agent; its bioactive compounds have also been identified as potential inhibitors against SARS-CoV-2 (Selim et al., 2022). Bioactive substances from *Pistacia atlantica* have shown promise as SARS-CoV-2 major protease inhibitors, indicating their potential use in COVID-19 studies (Nadjat et al., 2021). Furthermore, 1,2,3,4,6-pentagalloyl glucose from *Pistacia lentiscus* has shown inhibitory effects on SARS-CoV-2 replication and transcription processes, suggesting a potential role in battling the virus (Samandar et al., 2022). A recent study by Benguechoua et al. (2022) investigated the potential of quinic and digallic acids isolated from *Pistacia atlantica* Desf. leaves as inhibitors against the major protease and RNA-dependent RNA polymerase of SARS-CoV-2. The study found that these compounds showed promising inhibitory effects on critical viral enzymes, which could make them useful in combating the virus. This research highlights the growing relevance of the *Pistacia* species in fighting viral infections, which could lead to potential therapeutic breakthroughs. This growing body of research underscores the pharmacological significance of the species in the context of viral infections and warrants further investigation into their potential therapeutic applications. This cumulative evidence highlights the diverse and promising antiviral potential of different *Pistacia* species, emphasizing their role in exploring alternative treatments for viral infections, including SARS-CoV-2.

#### 3.2.13. *Silene* L

The *Silene* genus, belonging to the *Caryophyllaceae* family, comprises approximately 700 species globally, with significant diversity in Eurasia, especially in the Mediterranean and Southwest Asia (Mamadalieva et al., 2014). In Türkiye, the genus is widespread, with 175 identified species. Remarkably, 80 species are exclusive to Türkiye, showcasing an endemism ratio of approximately 46%. Additionally, 138 Silene species are found solely in Türkiye (Özbek and Uzunhisarcıklı, 2019).

Several *Silene* species are known for their culinary uses, with fresh green plant parts (excluding the root) of *S. vulgaris* being consumed as salads in the Sivas and Yozgat provinces of Türkiye (Özüdoğru et al., 2011). Traditional practices in Eastern Anatolia involve using *S. vulgaris* as a remedy for urinary inflammation (Zengin et al., 2018; Karpavičienė, 2022). A recent study focused on the inhibitory potential of extracts and secondary metabolites from *Silene* spp. (*Caryophyllaceae*) on SARS-CoV-2 replication (Orhan et al., 2009). The study highlighted the presence of biologically active substances, primarily in the root extracts, demonstrating efficacy against HSV-1 and parainfluenza viruses. Insights from the study suggest promising inhibitory effects at various effective dosages (Kazachinskaia et al., 2023), opening avenues for prospective therapeutic approaches against SARS-CoV-2.

As research into the broader pharmacological applications of *Silene* L. continues, it has emerged as a promising candidate for further investigations into potential antiviral properties and therapeutic benefits. The findings of Kazachinskaia et al. (2023) position *Silene* L. as a valuable subject for ongoing exploration in the quest for antiviral agents, particularly in combating SARS-CoV-2 and other related viruses. Future research endeavors can shed more light on the specific bioactive compounds responsible for the observed antiviral effects and their mechanisms of action.

#### 3.2.14. *Rheum* L

The *Rheum* genus, consisting of 60 species, is predominantly found in Asia, with Central Asia as a primary area for this variety. One notable species is *Rheum ribes* L., a perennial edible wild rhubarb plant found in Syria, Lebanon, Iran, Iraq, and Eastern Türkiye (Ekincialp et al., 2019). Belonging to the *Polygonaceae* family, this plant has a longstanding history of use as a food source, medicine, and supplemental medicine in Anatolia and other regions of the world. In places like Northern Iraq and Van in Türkiye, *R. ribes* roots are used to treat kidney disorders, diabetes, hypertension, and obesity (Keser et al., 2020). Additionally, the plant’s boiled roots are used in alternative herbal remedies to address various conditions such as gangrene, hypertension, mental fatigue, anorexia, and anemia (Abu-Irmaileh and Afifi, 2003). In Bitlis and other Southeastern regions of Türkiye, *R. ribes* aids digestion (Öztürk et al., 2007).

*R. ribes* is rich in phytocompounds, including polycyclic aromatic anthracene derivatives, which are important raw materials used in the pharmaceutical industry (Bati et al., 2020). It comprises over 250 bioactive substances, such as anthraquinones (emodin, chrysophanol, physcion, aloe-emodin, and emodin glycosides), anthrones, flavonoids, alkyl glucosides, and pylon stilbene (Bati et al., 2020). Several studies have demonstrated the antiviral potential of *Rheum* L., specifically *Rheum officinale* and *Rheum palmatum*. Chemicals like emodin and pyrogallol found in these plants have shown their antiviral capabilities, including the ability to suppress viruses like SARS-CoV-2, the virus causing COVID-19 (Kilic and Sener, 2022). These findings suggest that *Rheum* L. could be beneficial in developing novel medications with potential antiviral impacts by specifically targeting virus proteins.

Studies conducted by Bahramsoltani and Rahimi (2020), among others, have emphasized the effectiveness of traditional treatments, including those involving *Rheum* L., in treating SARS-CoV-2. This research is part of a growing body of evidence supporting the potential therapeutic role of *Rheum* L. in the context of COVID-19. Further research is needed to delve into the specific mechanisms of action and explore the development of medications based on the antiviral properties of *Rheum* L.

#### 3.2.15. *Prunus* L

The *Prunus* L. genus, comprising fruit-bearing shrubs and trees, is both botanically and economically significant, with an extensive distribution and a variety of fruits. In Türkiye, the *Rosaceae* family encompasses 58 native species and 37 genera, *Prunus* being one of them, totaling 297 species in the entire family (Browicz, 1972). One standout species is *Prunus amygdalus* var. amara (DC.) Focke, known as bitter almond. Originating in Central and Western Asia, particularly India, Iran, and Pakistan, this tree eventually emerged in the Mediterranean region and Türkiye (Rugini and Monastra, 2003; Şimşek et al., 2010).

Various species of the *Prunus* genus, particularly bitter almond kernels, contain the compound amygdalin, a cyanogenic glycoside. Amygdalin is noteworthy for its enzymatic hydrolysis, which transforms it into benzaldehyde, glucose, and hydrocyanic acid, contributing to the distinctive flavor and aroma of seeds and almond oil (El Bernoussi et al., 2020). Bitter almonds, when consumed at low concentrations, are associated with benefits such as chest softening, cough suppression, and pain relief; however, caution is warranted due to potential poisoning effects at high doses. Bitter almond oil is utilized for various purposes, including pain relief, diuretic properties, cough suppression, breast softening, and treating hemorrhoids and skin cracks (Xu et al., 2017).

Studies have indicated that the essential oil of *Prunus amygdalus* var. amara has antiviral effects against the bean yellow mosaic virus (BYMV) and *Pseudomonas* phage (Alkuwaiti et al., 2016). Moreover, almond oils have free radical scavenging ability and antioxidant activity, contributing to hepatoprotective effects and preventing liver damage (Feng et al., 2023). Almond kernels are a rich nutritional source of mono- and polyunsaturated fatty acids, tocopherols, and phenolic compounds (Geng et al., 2016). Recent studies utilizing network pharmacology have explored ephedra-bitter almonds’ active components and functional mechanisms for preventing and treating COVID-19 (Gao et al., 2020). Molecular docking studies revealed that the essential elements of ephedra-bitter almonds, including sitosterol, estrone, and stigmasterol, exhibited high binding activity to 3CL and ACE2 receptors, suggesting potential in developing novel COVID-19 therapeutics. However, it is crucial to interpret these findings cautiously, considering the study’s prospective nature and reliance on data mining.

#### 3.2.16. *Satureja* L

*Satureja* L., belonging to the *Lamiaceae* family, shares botanical connections with thyme and rosemary. Native to Central Asia, the Middle East, Southern and Southeastern Europe, and North Africa, this aromatic plant has diverse species (Moradi and Sadeghi, 2017). Notably, *Satureja parnassica* Heldr. and Sart. ex Boiss subsp. *sipylea* P. H. Davis is an endemic species in the Marmara region of Türkiye, particularly in Çanakkale and parts of Balıkesir (Satıl et al., 2002).

In rural therapeutics, *Satureja* is often consumed as an herbal tea and is locally known as “kokuluçay” or “dereçay”; it is believed to offer beneficial effects for coughs and gastrointestinal ailments (Tümen et al., 1992). In the Mediterranean region, *Satureja thymbra* is abundant, and its essential oil has historically been used in traditional spices and home remedies due to its antibacterial and diuretic properties. The oil exhibits strong antioxidant, antifungal, antibacterial, antiviral, and insecticidal activities (Fitsiou et al., 2016). The essential oil’s composition varies based on harvesting time, but two phenol monoterpenes, thymol and carvacrol, are consistently present in significant amounts (Fitsiou et al., 2016). Recent investigations have highlighted the potential of *Satureja* L. in the realm of antiviral effects, particularly against SARS-CoV-2. Ezaouine et al. (2022) shed light on *Satureja*’s distinctive attributes, emphasizing its multifaceted pharmacological properties, from antioxidant to antibacterial, and its potential antiviral activity. Başoğlu-Ünal et al. (2022) delved into *Satureja*’s potential as a potent inhibitor of the SARS-CoV-2 protease. Computational studies have revealed the specific phenolic compounds of *Satureja* and their promising binding activity against this critical viral enzyme. These studies position *Satureja* L. as a valuable resource for antiviral interventions, particularly against SARS-CoV-2. With a rich historical use and established pharmacological potential, *Satureja* emerges as a pivotal natural resource with significant implications for combating viral infections.

#### 3.2.17. *Cannabis* L

*Cannabis* L., originating in Central Asia, has a global presence due to its multifaceted uses. It provides nutrients, oil, fiber, and psychoactive compounds from its seeds, stems, and flowers, respectively. Hemp seed oil, extracted from *Cannabis sativa* L., serves various purposes, including making raw fuel and plastic (Tilkat et al., 2023). Historically, the Ottoman Empire used hemp for naval-related materials, such as rope, with notable production regions once found in Trabzon, Ordu, Canik, Aydın, İzmir, and Kastamonu (Akpınar and Nizamoğlu, 2019).

*Cannabis* has been utilized for therapeutic, industrial, and recreational purposes for thousands of years, with over 100 phytocannabinoid compounds identified. Active components like THC, CBD, CBG, CBC, and CBN are extensively researched for their potential medical applications. The endocannabinoid system plays a crucial role in mediating the positive effects of cannabinoids on various physiological functions (Türkölmez et al., 2023). Research has revealed the therapeutic benefits of cannabinoids found in *Cannabis* for viral disorders such as HIV and hepatitis (Lowe et al., 2017). In the context of SARS-CoV-2, cannabinoids, particularly CBD, have demonstrated various mechanisms beneficial in treating infections (Jahan and Onay, 2020; Onay et al., 2021). Vallée (2022) suggested that CBD may reduce the inflammatory response triggered by the SARS-CoV-2 spike protein, potentially acting as an antiviral drug by regulating cytokine production. Studies by Altyar et al. (2022) and van Breemen et al. (2022) build on the substantial evidence of *Cannabis sativa*’s ability to combat SARS-CoV-2. Various cannabinoids, including cannabidiolic acid, cannabichromanon, and cannabicyclolic acid, show strong binding affinities to vital viral proteins through molecular docking simulations. These interactions suggest a potential role in preventing viral proliferation, making cannabinoids potential candidates for treating COVID-19. Cannabinoid acids such as cannabigerolic and cannabidiolic acid showed significant benefits related to the SARS-CoV-2 spike protein, demonstrating the capability to prevent its viral entry and infection in human epithelial cells, even against SARS-CoV-2 variants.

Many studies have collectively illuminated the promising role of *Cannabis* L. and its derivatives in the battle against SARS-CoV-2. However, further research and clinical studies are essential to confirm their antiviral potential, safety, and efficacy in treating COVID-19. Previous findings have highlighted the potential of *Cannabis* L. as a valuable resource in ongoing efforts to combat SARS-CoV-2 and related viruses.

#### 3.2.18. *Alcea* L

*Alcea*, comprising 18 species in the Türkiye flora (Uzunhisarcıklı and Vural, 2009), has garnered attention for its diverse biological and medicinal activities. Among these, notable antiviral properties have been reported (Azab, 2016). Delgado-Paredes et al.’s (2021) study singled out *Alcea rosea* L., commonly known as hollyhock or “gül hatmi” in Turkish, as a noteworthy species with potential applications in addressing respiratory diseases, including COVID-19. Rich in flavonoid derivatives like quercetin and kaempferol, *Alcea rosea* L. has been identified as a promising candidate based on its composition. In a docking study, flavonoid derivatives found in *Alcea rosea* L. demonstrated potent inhibitory effects on the influenza H1N1 virus neuraminidase. This suggests a plausible capability to target the active site of the virus, indicating potential efficacy in combating influenza. The study positions *Alcea rosea* L. as a plant species meriting further exploration of its therapeutic potential, particularly in addressing influenza and potentially mitigating the effects of COVID-19. The flavonoid-rich composition of *Alcea rosea* L. adds to its appeal as a natural resource in the ongoing search for antiviral agents (Azab, 2016). The antiviral activities reported for *Alce*a, especially in *Alcea rosea* L., open avenues for future research to unravel its full potential in combating viral infections, including the possibility of addressing respiratory diseases such as COVID-19.

#### 3.2.19. *Pimpinella* L

*Pimpinella* is a plant genus in the *Apiaceae* family, prominently featuring *Pimpinella anisum* L., commonly known as anise, renowned for its aromatic herb properties (Shojaii and Fard, 2012). Anise, which flourishes in various regions of Türkiye, holds significance in medicinal and culinary practices. Anise seeds, prized for their medicinal and culinary applications, are integral to Turkish cuisine, imparting a sweet and aromatic flavor to dishes, drinks, and desserts. Anise tea, produced from the plant’s seeds, is a traditional drink, while anise oil is used in Turkish medicine for its potential antimicrobial and digestive properties.

*Pimpinella anisum* is historically associated with alternative and conventional medicine due to its perceived antiviral properties against infections (Soussi et al., 2023). However, further research is needed to precisely delineate its antiviral effects. Recent studies have suggested the potential of *Pimpinella anisum* L. in preventing and treating COVID-19 infections, particularly in moderate cases. The essential oil extracted from *Pimpinella anisum* L. medicinal plants has been investigated for its inhibitory effects on the crucial viral protein 3CL protease of SARS-CoV-2 (Nasır and Yabalak, 2021). Hendi et al. (2022) explored the creation of zinc oxide nanoparticles using a mixture of olive and black seed essential oils, incorporating extracts from *Nigella sativa* and *Pimpinella anisum* L. The study revealed promising bioactive chemicals in these extracts’ nanostructure, highlighting the potential of *Pimpinella anisum* in developing effective COVID-19 inhibitors. However, thorough experimental validation and clinical trials are essential to confirm its efficacy.

Hasan et al. (2020) investigated phytochemicals from *Pimpinella anisum* as potential inhibitors of the critical enzyme 3C-like protease crucial for SARS-CoV-2 replication. Eleven phytochemicals exhibited notable binding energies to the protease, suggesting a potential to reduce its proteolytic activity. These findings warrant further exploration in controlled laboratory settings and real-world applications, positioning phytochemicals from *Pimpinella anisum* L. as possible therapeutic agents against SARS-CoV-2. *Pimpinella anisum* L. emerges as a plant of interest with multifaceted applications, from its culinary use to potential antiviral interventions. Ongoing research holds promise to uncover its full therapeutic potential and contribute to the global effort against viral infections, including COVID-19.

#### 3.2.20. *Echinacea* L

*Echinacea*, a genus in the daisy family, comprises herbaceous flowering plants, including 10 different species of coneflowers. Among these, *Echinacea purpurea*, known as purple coneflower, has garnered attention for its potential antiviral qualities, making it a subject of interest in the fight against COVID-19 (Burlou-Nagy et al., 2022). This research has explored the antiviral potential of *Echinacea purpurea*, particularly against certain SARS-CoV-2 variants of concern (VOCs). Vimalanathan et al. (2022) conducted a study demonstrating that the hydroethanolic extract of *Echinacea purpurea* L. roots and herb, Echinaforce, exhibited remarkable inhibitory effects against a range of VOCs, including alpha, beta, gamma, delta, Scottish, and eta variants. This extract showed significant potential in restricting the propagation of these variants, presenting a valuable contribution to preventive measures.

Nicolussi et al. (2022) highlighted *Echinacea*’s potential based on randomized controlled trials. These trials revealed that *Echinacea* extract was crucial in reducing the incidence of enveloped virus infections, including various coronaviruses, in adults and children. The extract reduced viral loads and mitigated respiratory symptoms during coronavirus infections. This positions *Echinacea purpurea* L. as a promising candidate in reinforcing the body’s defenses against COVID-19 and respiratory diseases.

Molecular dynamics analyses conducted by Vimalanathan et al. (2022) provided valuable insight into the mechanism of action of *Echinacea* extract. The phytochemical components of the extract were shown to consistently bind to key viral spike proteins and the host cell protease TMPRSS2. This suggests potential virucidal activity, inhibiting vital processes such as viral cell entry and membrane fusion. *Echinacea purpurea* emerges as a multifaceted botanical specimen in the battle against SARS-CoV-2. Its demonstrated ability to target a range of VOCs and interfere with viral entry mechanisms makes this botanical extract a pivotal player in combating COVID-19. Although it is a promising extract, further detailed tests and research are essential to establishing its efficacy and safety in the context of viral infections.

#### 3.2.21. *Matricaria* L

*Matricaria* is a genus of flowering plants in the *Asteraceae* family, belonging to the chamomile tribe. *Matricaria chamomilla* L., commonly known as chamomile, is recognized for its potent antibacterial and antioxidant properties (Parham et al., 2020). As highlighted by Singh et al. (2011), chamomile contains various beneficial compounds, including flavonoids, terpenoids, phenolic compounds, apigenin, and matricin. The presence of flavonoids contributes to its antioxidant properties. The pharmacological effects of chamomile are widespread, encompassing antioxidative, antibacterial, antiinflammatory, antifungal, analgesic, anticancer, antihypoglycemic, antistress, antihypertensive, and hepatoprotective qualities (Parham et al., 2020). However, it should be mentioned that the antiviral effects of chamomile may not extend to HSV-2 (Allahverdiyev et al., 2013).

Chamomile has gained recent attention in the context of the fight against COVID-19. A molecular docking study conducted by Habibzadeh and Zohalinezhad (2021) explored the antiviral potential of *Matricaria chamomilla* L. against SARS-CoV-2. The study identified two significant flavonoid components of chamomile, apigenin and luteolin, as strong inhibitors of the primary protease protein of SARS-CoV-2. This suggests that chamomile could effectively limit the entry of SARS-CoV-2 into human cells. Moghaddam et al. (2021) delved into the effects of chamomile extract on coronavirus infections. With over 120 biologically active compounds, including flavonoids like apigenin and luteolin, chamomile demonstrated antiviral activity. The study highlights chamomile’s potential as a therapeutic agent against coronaviruses, including SARS-CoV-2.

The historical use of chamomile in traditional Anatolian and Persian medicine and its recognition by medical scholars like Avicenna and Rhazes underscore its significance in treating respiratory diseases. Many studies have shed light on the promising antiviral properties of *Matricaria chamomilla*, particularly in the context of SARS-CoV-2. Findings from this research provide a basis for further exploration of chamomile’s potential role as a preventive or therapeutic agent in managing COVID-19 and other coronavirus infections (Habibzadeh and Zohalinezhad, 2021)

#### 3.2.22. *Aloe* L

The *Aloe* genus comprises over 650 species of flowering succulent plants, originally native to Southern and tropical Africa, Madagascar, Jordan, the Arabian Peninsula, and various Indian Ocean islands. Some species, including *Aloe vera* (L.) Burm.f., have expanded beyond their original habitats and are now found in the Mediterranean region and Türkiye (Davis, 1984).

*Aloe vera*, a popular variant in the *Aloe* genus, is a perennial herb native to the Mediterranean region, the Arabian Peninsula, India, China, and Eastern Africa. Recognized by its juicy leaves, *Aloe vera* is known as “sarısabır” in Turkish (yellow aloe) in Anatolia due to its primarily yellow blossoms. It grows naturally in the southwest coast of Türkiye and in the Demre neighborhood of Antalya (Tunçay and Kaya, 2021). *Aloe vera* has been the subject of antiviral investigations, demonstrating activities against various viruses in earlier studies (Mpiana et al., 2020a, 2020b). Recent research focused on identifying *Aloe vera* compounds with promising in silico binding abilities against SARS-CoV-2 NSP-16. While these results suggest the potential of *Aloe vera* chemicals as inhibitors, further in vitro and in vivo research is necessary to ascertain their precise antiviral activities and potential application in treating SARS-CoV-2 (da Silva et al., 2020). Mpiana et al. (2020a) explored *Aloe vera*’s antiviral properties against several viruses, including SARS-CoV-1. Their study investigated the potential contribution of specific plant compounds and minerals, such as zinc, to the antiviral activities of *Aloe vera*. While its efficacy against COVID-19 is promising, additional molecular docking and clinical trials are required (Mpiana et al., 2020b). A study by Españo et al. (2022) delved into the potential of *Aloe vera* chemicals, especially aloe emodin and acemannan, as antivirals and immunomodulators for treating viral illnesses. The research suggests that these extracts could be utilized to develop antiviral medications and preventive measures. Additionally, the authors proposed using *Aloe vera* chemicals as adjuvants in viral vaccinations, emphasizing their potential in medical applications. Despite these and other possibilities, challenges exist in using aloe chemicals, particularly those from *Aloe vera*, in clinical settings. The present study underscores the untapped potential of *Aloe vera* in antiviral medicinal and vaccine development, urging further exploration and research into its capabilities.

#### 3.2.23. Lemon balm (*Melissa officinalis* L.)

Lemon balm (*Melissa officinalis* L.) is a perennial herb native to Southern Europe, North Africa, and Anatolia. It belongs to the *Lamiaceae* family and the *Melissa* genus. Thriving in diverse habitats such as open forests, shrublands, rocky slopes, riverbanks, barren areas, and roadsides, lemon balm can be found at altitudes ranging from sea level to 1800 m (Kokkini et al., 2003). Lemon balm has significant antiviral potential, extending to viruses such as influenza A (H9N2) and SARS-CoV-2 (Sampangi-Ramaiah et al., 2020). Specific compounds in *Melissa officinalis* have been identified as promising in inhibiting the M^pro^ of SARS-CoV-2, offering a prospective avenue for novel antiviral interventions (Elekofehinti et al., 2021).

Prasanth et al. (2021) conducted a comprehensive in silico analysis, highlighting specific phytoconstituents from lemon balm with significant binding affinity and stability to SARS-CoV-2’s main protease and spike protein. Compounds such as luteolin-7-glucoside-3’-glucuronide, melitric acid-A, and quadranoside-III were identified as potential candidates, suggesting the need for further exploration in vitro and in vivo studies (Prasanth et al., 2021). A recent review by Behzadi et al. (2023) emphasized the diverse antiviral potential of *Melissa officinalis* L. The plant exhibits efficacy against a range of viruses, including SARS-CoV-2, HSV, and HIV. Its mechanisms of action, such as viral binding inhibition and interference with essential viral proteins, make it a promising complementary antiviral drug. The present study emphasizes the importance of a deeper understanding of lemon balm’s mechanisms and clinical applications.

With its rich history and widespread availability, lemon balm is a natural antiviral agent with the potential to contribute to developing novel interventions against viral infections. Further research, including in vitro and in vivo studies, is crucial for validating its efficacy and exploring its application in clinical settings.

#### 3.2.24. *Ocimum* L

*Ocimum basilicum* L., commonly known as basil, belongs to the *Lamiaceae* family; it is part of the *Ocimum* genus and comprises over 150 recognized species. In addition to its widespread use in gastronomy, basil has long been associated with traditional medicine, particularly in addressing respiratory and gastrointestinal ailments. Research indicates its potential as a versatile agent, demonstrating antiviral, antibacterial, and antifungal properties (Yasmin et al., 2020). Researchers studying its impact on the BVD virus found that the essential oils rich in monoterpenes derived from *Ocimum basilicum* displayed direct interactions with viral particles, resulting in a significant reduction in virus count observed through plaque assays (Chávez-González et al., 2016). In a recent study, the focus shifted to investigating the potential antiviral activity of polyphenolic compounds found in *Ocimum basilicum* against the primary protease of SARS-CoV-2. Compounds like apigenin-7-glucuronide and dihydrokaempferol-3-glucoside exhibited robust interactions, suggesting significant potential as antiviral agents. ADMET (absorption, distribution, metabolism, excretion, and toxicity) assessments and their similarity to other drugs further corroborated the promise of these compounds. The study showed that these chemicals merit further investigation for the development of potential therapies for COVID-19 (Kurnia et al., 2023). Beyond its culinary applications, basil stands out for its promising antiviral properties. From its demonstrated efficacy against the BVD virus to its potential activity against SARS-CoV-2, *Ocimum basilicum* L. is a natural resource worthy of thorough exploration in developing antiviral therapies. Ongoing research in this direction promises to unravel valuable insights for treating various viral infections, including those caused by emerging viruses such as SARS-CoV-2.

## 4. Conclusion and future perspectives

The COVID-19 pandemic has spurred intensified efforts to seek effective antiviral agents, particularly in developing vaccines and therapeutic drugs. Anatolian medicinal plants, rich in biodiversity and steeped in tradition, have emerged as a promising frontier for discovering novel antiviral medications. This present analysis has gathered information on 24 taxa of plant species from Anatolia, unveiling their traditional uses and highlighting their inherent antiviral properties. Spanning from *Prunus* L. to *Ocimum* L., these plants showcase a variety of compounds with the potential for antiviral effects.

ATM, deeply rooted in the region’s history, is a valuable repository of knowledge. The array of plant species and their associated traditional uses provide a robust foundation for studying and potentially developing novel antiviral compounds. While the gathered information is promising, there is a critical need for further research to fully unlock the potential of medicinal plants from Anatolia. Rigorous animal experiments, clinical trials, and case reports are imperative to validate antiviral efficacy and ensure the safety of herbal-based medicine for human use. Challenges in standardization, dosage determination, and safety assessment loom large but present an opportunity. Overcoming these challenges allows us to tap into the extensive knowledge and biodiversity of Anatolian medicinal plants to develop antiviral drugs. This review is a comprehensive resource for scientists and researchers developing next-generation, broad-spectrum antiviral drugs. The amalgamation of knowledge derived from ATM holds the potential to inspire innovative solutions against the virulence of various viruses, including SARS-CoV-2.

In conclusion, exploring Anatolian medicinal plants for antiviral development is very promising. Integrating traditional wisdom with modern scientific research can lead to the discovery of new compounds and drugs with potential applications against viral infections. Collaborative efforts between botanists, pharmacologists, and clinicians will be essential in advancing these findings from theoretical promise to practical, clinically validated solutions. As the quest for antiviral agents persists, Anatolian medicinal plants are a testament to nature’s potential in addressing global health challenges.

## Figures and Tables

**Table:** Partial list of common plant species in Türkiye, particularly Anatolia, known for their antiviral properties and application in TAM.

Latin name	Common or regional plant name (English, Turkish)	Antiviral compound(s)	Use in conventional medicine with institutional approval	References
*Allium sativum Allium tuncelianum*	Garlic, sarımsak, Tunceli sarımsağı	Alliin (S-allyl-L-cysteine sulfoxide), diallyl sulfide	(generally antiviral) Adenovirus-3, coronavirus (CoV); SARS-CoV	Aykur et al. ( 2020); Akram et al. (2021)
*Allium cepa*	Garden onion, soğan	Quercetin, kaempferol zalcitabine, allicin, and ribavirin	Adenovirus	Sharma (2019)
*Salvia officinalis Salvia suffruticosa*	Sage, adaçayı	Safficinolide and sage one	VSV (vesicular stomatitis virus), SARS-CoV	Yılmaz et al. (2017); Elebeedy et al. (2021)
*Sideritis brevibracteata*	Mountain tea, dağ çayı	Linearol	HIV	Abeshi et al. (2017)
*Thymus vulgaris*	Common thyme, adi kekik	Carvacrol, thymol	Herpes simplex virus 1 and 2, Newcastle disease virus, SARS-CoV-2	Javed et al. (2021)
*Origanum vulgare*	Common oregano, kekik	Carvacrol, linoleic acid	Influenza virus; Murine nanoviruses	Gilling et al. (2014); Sargin (2021)
*Mentha spicata*	Spearmint, bahçe nanesi	Mentofin	Avian influenza virus	Barbour et al. (2010)
*Rosmarinus officinalis*	Rosemary, biberiye	Rosmarinic acid	HSV-1/HSV-2	AL-Megrin et al. (2020)
*Pistacia vera, Pistacia lentiscus*	Pistachio, Antep fıstığı, mastic/lentisk, sakız	Linoleic acid, palmitic acid	HSV-1, parainfluenza, SARS-CoV-2	Onay et al. (2021)
*Silene vulgaris*	Bladder campion, gıvışgan otu	Stem extract	HSV-1, parainfluenza	Orhan et al. (2009)
*Rheum ribes*	Syrian rhubarb, ışkın	Ethanol extract	HSV	Tartik et al. (2015)
*Ecballium elaterium*	Squirting cucumber, acı hıyar	Cucurbitacin B	HSV-1	Hassan et al. (2017)
*Prunus amygdalus* var. *amara*	Bitter almond, acı badem	Amygdalin	BYMV, *Pseudomonas* phage	Alkuwaiti et al. (2016)
*Satureja parnassica Satureja thymbra*	Savory, kekik	Carvacrol, thymol	CMV, HSV-1, parainfluenza type-3	Fitsiou et al. (2016)
*Cannabis sativa*	Cannabis, kenevir	THC and CBD	HIV-1, Hepatitis B and Hepatitis C, SARS-CoV-2	Lowe et al. (2017); Jahan and Onay (2020); Onay et al. (2021)
*Aloe vera* (L.) Burm.f.	*Aloe vera*, sarısabır	Quercetin, catechin hydrate, kaempferol	SARS CoV-2 M^pro^ inhibition; quercetin inhibits the SARS CoV-2 ACE2 receptor	Choi et al. (2019); Solnier and Fladerer (2020)
		Feralolide	Prevents the replication of SARS-CoV-2 by blocking the main protease (3CL^pro^)	Mpiana et al. (2020a)
*Cyperus rotundus* L.	Nutgrass, topalak	Humulene epoxide	PL^pro^, 3CL^pro^, RdRp, and the spike glycoprotein are four of the SARS-CoV-2 virus’s target proteins	Amparo et al. (2021)
*Ocimum basilicum* L	Basil, fesleğen	Apigenin-7-glucuronide and dihydrokaempferol-3-glucoside	SARS-CoV-2 ACE2 receptor and 3CL^pro^ inhibition	Kurnia et al. (2023)

## Footnotes

1 WHO (2020). Coronavirus disease (COVID-19) pandemic [online]. Website: https://www.who.int/news/item/30-01-2020-statement-on-the-second-meeting-of-the-international-health-regulations-(2005)-emergency-committee-regarding-the-outbreak-of-novel-coronavirus-(2019-ncov) [accessed 12 August 2023].

2 WHO dashboard (2023). COVID-19 Weekly Epidemiological Update [online]. Website: https://covid19.who.int [accessed 12 August 2023].
